# Open Surgical Retrieval of a Perforated Inferior Vena Cava Filter: A Case Report

**DOI:** 10.7759/cureus.86830

**Published:** 2025-06-26

**Authors:** Eiji Nakamura, Satoru Tobinaga, Tomofumi Fukuda, Hirotoshi Tsuru, Hiroshi Yasunaga

**Affiliations:** 1 Department of Cardiovascular Surgery, St. Mary’s Hospital, Kurume, JPN

**Keywords:** aortic injury, inferior vena cava filters, late complications, perforation, pulmonary embolism, surgical retrieval

## Abstract

Inferior vena cava (IVC) filters are used to prevent fatal pulmonary embolism (PE) and may be associated with late-stage complications as filter hooks may deviate over time. Prolonged implantation and challenges in retrieving these filters can lead to damage to the surrounding organs. In this report, we described a case in which a thrombus in the IVC filter made retrieval difficult. Following 1.5 years of implantation, the IVC filter had perforated, necessitating surgical retrieval due to the risk of aortic injury.

A 56-year-old woman presented with right lower extremity venous thrombosis and asymptomatic PE; anticoagulation therapy with edoxaban 60 mg once daily was started after the placement of an IVC filter. On the 10th day, computed tomography (CT) imaging revealed the development of venous thrombosis leading to thrombosis and occlusion of the IVC, which complicated filter retrieval. CT findings 1.5 years post-placement showed thrombus lysis and recanalization due to anticoagulation therapy with edoxaban 60 mg once daily. The patient was asymptomatic. However, filter perforation outside the IVC was observed, with one of the six perforated filter hooks located proximally to the abdominal aorta, raising concerns of potential aortic injury. Open surgical retrieval of the perforated IVC was performed under general anesthesia, with no evidence of aortic injury. The perforated hook was retrieved from outside the IVC, followed by a retrieval of the remaining filter body through venotomy. The patient's postoperative condition was stable; she was discharged without complications, and anticoagulation therapy with edoxaban 60 mg once daily was continued for six months postoperatively.

IVC filter perforation is a common late complication associated with filter use. Currently, no clear consensus exists regarding these treatment strategies, and further research and case studies are warranted.

## Introduction

Inferior vena cava (IVC) filters are used to avoid fatal pulmonary embolism (PE) in patients with deep vein thrombosis (DVT) and are strongly recommended, especially in situations where anticoagulation is not possible [1,2]. IVC filters are used not only when anticoagulant therapy is difficult, but also for PE and proximal DVT in cases where PE recurs or worsens even under anticoagulant therapy, or where re-embolization of residual thrombus can lead to fatal PE. Data from registries in Japan between 2009 and 2010 indicated an IVC filter insertion rate of approximately 40% for symptomatic PE, DVT, and asymptomatic acute proximal DVT [3]. There are two types of filters available in Japan: retrievable and temporary. The retrievable filter is approved for use as a permanent implantation when retrieval is difficult. Usually, a minimally invasive endovascular retrieval is performed. In most cases, venous access is via the right internal jugular vein, and the filter head is grasped with a dedicated retrieval kit and advanced through the introducer sheath to retrieve it into the sheath. However, the efficacy of IVC filters in preventing fatal PE is only seen in early onset stages, with no significant difference in incidence or survival during remote stages. A higher proportion of late complications, such as venous thrombosis, are observed at remote stages, suggesting that the early PE prevention effect of IVC filters is offset by increased late complications [1,2]. Although many IVC filters are now retrievable, cases of long-term implantation still exist [4].

In addition to venous thrombosis, a significant complication of long-term IVC filter retention is the perforation of the filter. This is a structural complication, with a high rate of approximately 19% [5]. Currently, the optimal treatment strategy for cases of IVC filter perforation remains controversial, and treatment consensus among practitioners is lacking.

We report a case in which an IVC filter had perforated 1.5 years after placement, and the perforated IVC filter was surgically retrieved because of its potential to cause aortic injury.

## Case presentation

A 56-year-old woman presented to her previous physician with swelling in the right lower extremity. Her consciousness was clear, and her vital signs were blood pressure of 114/64 mmHg, pulse rate of 87 beats/min, respiratory rate of 18 breaths/min, and body temperature of 36.1°C. She had no hypertension but was complicated by diabetes mellitus, well controlled with diet and exercise therapy only. After a thorough examination, asymptomatic PE and DVT of the right lower extremity were revealed, and anticoagulation therapy with edoxaban 60 mg once daily was initiated after the placement of an ALN IVC filter (ALN Implants Chirurgicaux Ghisconaccia, France). Physical examination revealed no other notable findings except severe obesity (159 cm, 93 kg, body mass index: 37) and no congenital thrombophilia.

**Figure 1:**
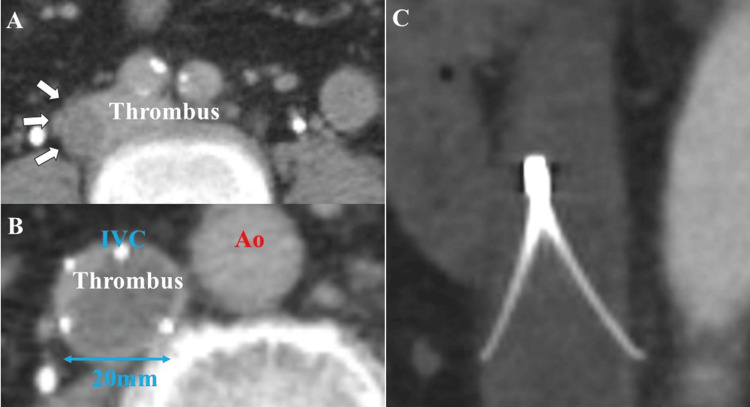
CT findings 10 days after IVC filter placement (A-B) CT findings, 10 days after IVC placement, show right iliac vein thrombosis (white arrows) extending proximally, and the IVC is occluded with a venous thrombus. The diameter of the IVC (blue double-headed arrow) is 20 mm. (C) The IVC filter is located in the IVC without any perforation. CT: computed tomography; IVC: inferior vena cava; Ao: aorta

Computed tomography (CT) performed 10 days after IVC placement showed that iliac vein thrombosis had developed proximally, resulting in thrombus filling of the filter and occlusion of the IVC with a diameter of 20 mm (Figures 1A-1B). It was determined that retrieval of the IVC filter would be difficult, and a decision was made to retain the filter for long-term anticoagulation therapy. No perforations were observed (Figure 1C).

**Figure 2:**
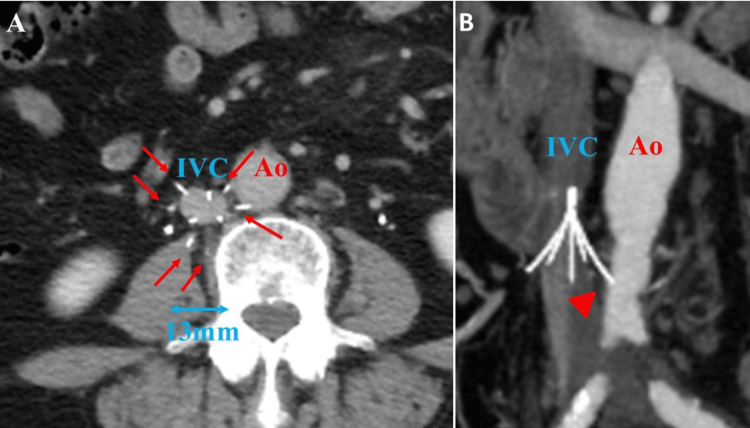
CT findings after 1.5 years of IVC filter placement (A) CT findings 1.5 years after placement of the IVC filter show that the IVC was recanalized and that six of the nine struts of the IVC filter (red arrows) had perforated outside the IVC. The IVC diameter (blue double-headed arrows) is reduced to 13 mm. (B) Struts perforating the left side of the IVC (red arrowhead) were located near the abdominal aorta. Ao: aorta; CT: computed tomography; IVC: inferior vena cava

CT findings 1.5 years after IVC filter placement revealed that the occluded IVC, including previously recognized iliac venous thrombus and PE, was dissolved and recanalized with continued anticoagulation therapy. The diameter of the IVC was reduced to 13 mm (Figure 2A). In addition, the filter struts clearly deviated from the IVC, confirming perforation (Figure 2B).

The ALN IVC filter had six struts with hooks and three struts without hooks, forming nine legs. Upon CT imaging, only the three struts without hooks remained in the IVC, and all six struts with hooks deviated outside the vessel (Figure 2A). There were two deviations each on the left, right, and dorsal sides. On the right side, the duodenum and ureter were present but separated. On the dorsal side, the struts had deviated close to the iliopsoas muscle and vertebral body but were undamaged. On the left side, an approximately 9 mm deviation was observed, entering into the dorsal adventitia of the aorta. Surgical retrieval of the IVC filter was scheduled due to the possibility of aortic injury. The patient was referred to our hospital.

**Figure 3:**
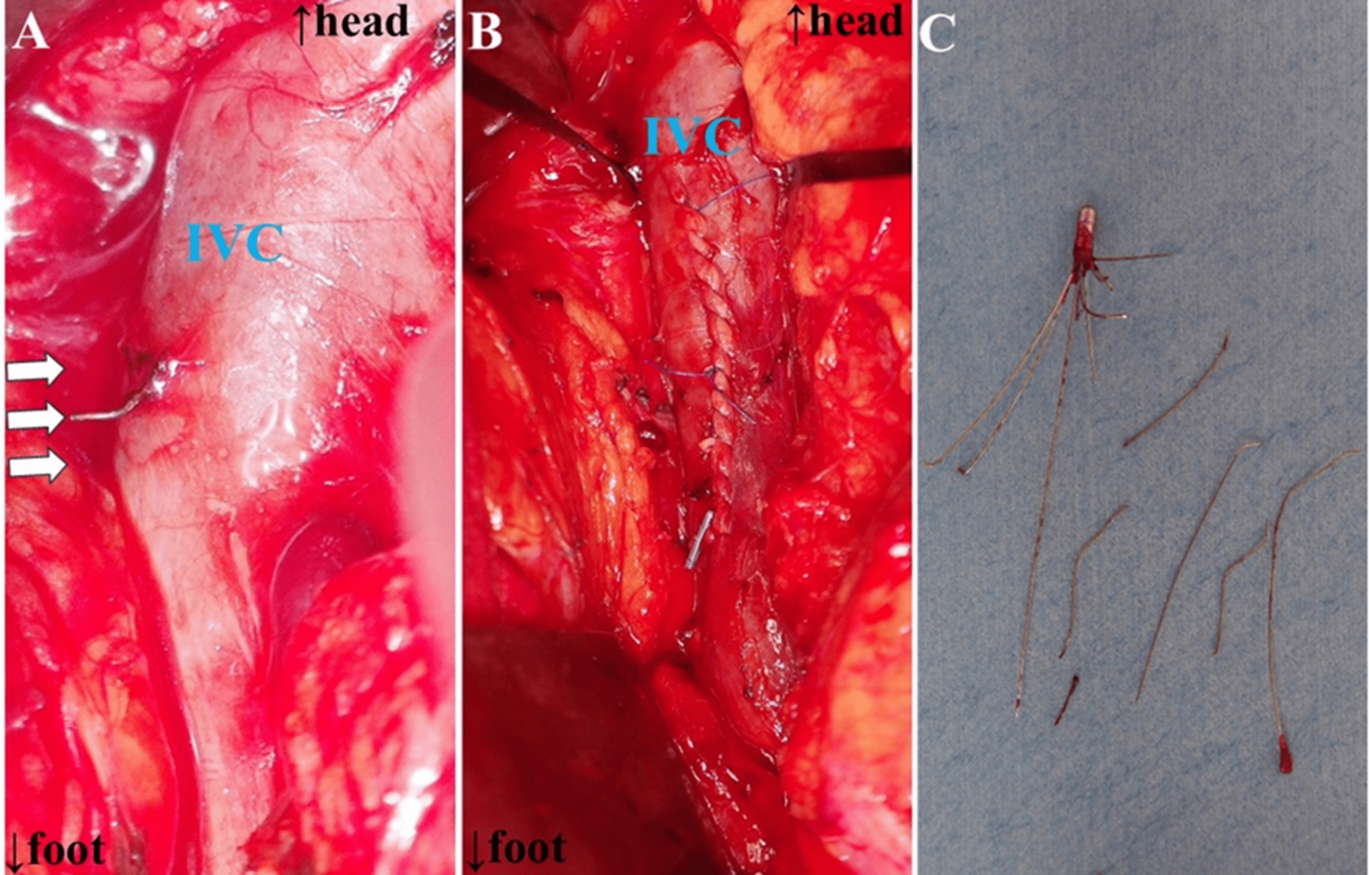
Intraoperative photograph (A) Intraoperative findings show the filter struts (white arrows) perforated outside the IVC. The deviated filter struts were first cut with a cutter outside the IVC, and the remaining filter body was retrieved after venotomy. (B) The incised IVC was closed with simple suture closure using a continuous suture. The vessel clip was on a small branch. The image C shows the extracted filter strut and filter body. IVC: inferior vena cava

Open surgical retrieval of the perforated IVC filter was performed under general anesthesia through median laparotomy. Upon laparotomy, it was observed that the hooks had completely deviated from the IVC, as shown on the CT findings (Figure 3A), but no aortic injury was observed. The hooks outside the IVC were first cut off with a cutter, and the remaining filter was retrieved after venotomy. The incised IVC was closed using a continuous suture with a 4-0 Prolene suture (Ethicon Inc., Somerville, NJ, USA) (Figures 3B-3C).

The patient exhibited an uneventful clinical course and was subsequently discharged from the hospital. Postoperative anticoagulation therapy (edoxaban 60 mg once daily) was continued for six months and then discontinued. No recurrence of venous thrombosis has been observed since then.

## Discussion

Previous reports have shown that IVC filters are effective in preventing PE in the early stages; however, their effectiveness in late-stage complications, such as venous thrombosis, is reduced [1,2]. Those findings show that the IVC filter had no advantage in preventing PE under anticoagulant therapy. Therefore, if anticoagulation is possible, IVC filter placement should be avoided, where late complications can be problematic.

In addition to venous thrombosis being a late-stage complication of long-term IVC filter implantation, the structural abnormality of IVC filter perforation, as in the present case, could be problematic. The rate of IVC filter perforation is as high as 19% in individuals with implants [5]. However, the symptomatic rate of perforated cases is as low as 8%, with many patients being asymptomatic to IVC filter perforation. A review found that in a total of 9002 patients with long-term IVC filter implantation, filter perforation was observed in 19% of the patients. In addition, a review found organ damage in 19% of perforated cases. Organ damage occurred in the duodenum, vertebral body, and aorta, necessitating therapeutic interventions in half of the cases and endovascular retrieval in 40% of the perforations. However, only 4% of the cases were surgically removed, and most of the reported cases appeared to have undergone endovascular retrieval. On the other hand, there have been reports of failed endovascular retrieval resulting in perforation of the duodenum due to subsequent thrombectomy with a residual fragment of the filter fracture [6].

The mechanism of the IVC filter perforation is generally considered to be a result of the presence of a hook for fixation, which must penetrate the vein wall slightly. Aortic pulsation and respiratory motion lead to repeated trauma to the vena cava wall; this is thought to be positively correlated with the length of indwelling time [7]. In the present case, we suspect that the mechanism of IVC perforation by the filter may also involve a change in IVC diameter after thrombus occlusion. As reported in a previous study [8], the risk of IVC filter perforation has been shown to increase with time, especially in relatively small IVCs. In our case, the IVC diameter had narrowed from 20 mm immediately after implantation to 13 mm when the perforation was detected 1.5 years later. Most venous thrombi adhere to the vessel wall and become organic within a few days due to inflammation. Subsequently, they are believed to cause regression owing to their fibrinolytic and angiogenic effects. This report suggests that the veins tend to shrink during chronic inflammation after thrombotic occlusion.

Data from registries in Japan between 2009 and 2010 indicated an IVC filter insertion rate of approximately 40% for symptomatic PE, DVT, and asymptomatic acute proximal DVT; more filters were implanted in Japan than in other countries [3]. In recent years, the establishment of anticoagulant therapy and guidelines has expected to decrease in the number of new cases of IVC filter implantation in Japan, as in other countries [9], but many IVC filters have been implanted in the past and there are thought to be a certain number of cases of difficult to retrieval. Therefore, easy implantation of IVC filters in cases with late complications such as venous thrombosis or perforation should be avoided. Filters should only be used to prevent acute pulmonary thromboembolism and should be removed as soon as possible.

The ALN filter should be retrieved within 10 days of implantation according to the manufacturer’s instructions; it has been reported that ALN filters can be retrieved even in cases of long-term implantation [4]. Furthermore, it has been reported that perforated IVC filters can be removed relatively safely and that filter perforation should not be considered ineligible for retrieval [10].

A major reason for the difficulty in filter retrieval is that the head of the filter is often snared due to its embedded head and tilt [5]. Dinglasan et al. showed that in 48 cases of difficult filter retrieval, 90% of patients had filters retained within the vessel wall [11]. When standard techniques fail, advanced retrieval techniques are often used, including the sling method and endobronchial forceps [12-14]. The retrieval success rate has significantly improved using these advanced techniques.

Although this is a retrospective case study with limitations, the following observations were that open surgical retrieval of the perforated IVC filter could have been avoided if close follow-up had been performed and retrieval had been attempted once the intrafilter thrombus had dissolved (even though intrafilter thrombus formation made retrieval difficult). Open surgical retrieval may have been considered when endovascular retrieval is not feasible. There was also a report of intrafilter thrombus dissolution 15 days after initiation of apixaban in combination with consolidation therapy, indicating the usefulness of direct oral anticoagulants [15] and that CT follow-up should have been performed at least every month to confirm thrombus dissolution during the acute phase of this case. Retrievable filters are known to have a higher complication rate than permanent filters, and the insertion of retrievable filters in younger patients, who are considered to have a longer prognosis, should be avoided as much as possible [16]. In addition, when a filter is implanted, efforts should be made to retrieve as much as possible, since the late complication rate increases over time with long-term IVC filter implantation. On the other hand, the natural prognosis of asymptomatic cases with perforated IVC filters and the safety and prognostic efficacy of surgical and endovascular retrieval for asymptomatic cases are currently unclear and require further study.

## Conclusions

Filter perforation is common after long-term implantation. When it is judged that filter retrieval is difficult due to trapped thrombus in the filter in the early stage after IVC filter implantation, it is advisable to continue anticoagulation therapy with direct oral anticoagulants and follow CT at close intervals, such as every month, to keep a close watch on when the thrombus dissolves and to attempt endovascular retrieval before late complications such as perforation occur and to avoid prolonged retention of the filter as much as possible.

The remote prognosis and optimal treatment of IVC filter perforations from long-term placement are currently unknown and require further study.
